# Nanoradiopharmaceuticals: Design Principles, Radiolabeling Strategies, and Biomedicine Applications

**DOI:** 10.3390/pharmaceutics17070912

**Published:** 2025-07-14

**Authors:** Andrés Núñez-Salinas, Cristian Parra-Garretón, Daniel Acuña, Sofía Peñaloza, Germán Günther, Soledad Bollo, Francisco Arriagada, Javier Morales

**Affiliations:** 1 Universidad Andres Bello, Escuela de Química y Farmacia, Facultad de Medicina. 8370016. Santiago. Chile; andres.nunez@unab.cl (A.N.-S.); cristian.parra@unab.cl (C.P.-G.); 2 Departamento de Ciencias y Tecnología Farmacéutica, Facultad de Ciencias Químicas y Farmacéuticas, Universidad de Chile, Santiago 8380492, Chile; daniel.acuna.a@ug.uchile.cl (D.A.); sofia.penaloza@ug.uchile.cl (S.P.); 3 Departamento de Química Orgánica y Fisicoquímica, Facultad de Ciencias Químicas y Farmacéuticas, Universidad de Chile, Santiago 8380492, Chile; ggunther@ciq.uchile.cl; 4 Departamento de Química Farmacológica y Toxicológica, Facultad de Ciencias Químicas y Farmacéuticas, Universidad de Chile, Santiago 8380492, Chile; sbollo@ciq.uchile.cl

**Keywords:** nanoradiopharmaceuticals, radiopharmaceuticals, radiolabeled nanoparticles, theranostics, nanomedicine, molecular imaging

## Abstract

Nanoradiopharmaceuticals integrate nanotechnology with nuclear medicine to enhance the precision and effectiveness of radiopharmaceuticals used in diagnostic imaging and targeted therapies. Nanomaterials offer improved targeting capabilities and greater stability, helping to overcome several limitations. This review presents a comprehensive overview of the fundamental design principles, radiolabeling techniques, and biomedical applications of nanoradiopharmaceuticals, with a particular focus on their expanding role in precision oncology. It explores key areas, including single- and multi-modal imaging modalities (SPECT, PET), radionuclide therapies involving beta, alpha, and Auger emitters, and integrated theranostic systems. A diverse array of nanocarriers is examined, including liposomes, micelles, albumin nanoparticles, PLGA, dendrimers, and gold, iron oxide, and silica-based platforms, with an assessment of both preclinical and clinical research outcomes. Theranostic nanoplatforms, which integrate diagnostic and therapeutic functions within a single system, enable real-time monitoring and personalized dose optimization. Although some of these systems have progressed to clinical trials, several obstacles remain, including formulation stability, scalable manufacturing, regulatory compliance, and long-term safety considerations. In summary, nanoradiopharmaceuticals represent a promising frontier in personalized medicine, particularly in oncology. By combining diagnostic and therapeutic capabilities within a single nanosystem, they facilitate more individualized and adaptive treatment approaches. Continued innovation in formulation, radiochemistry, and regulatory harmonization will be crucial to their successful routine clinical use.

## 1. Introduction

Radiopharmaceuticals are defined as molecules containing a radionuclide that are used for the diagnosis or treatment of diseases [1]. They are central to nuclear medicine, leveraging the unique physical properties of radioactive isotopes while retaining the same chemical behavior as their stable counterparts. Nuclear imaging exploits the absence of naturally radioactive biomolecules in the body, enabling a high-contrast visualization of radiopharmaceuticals. Based on the radionuclide, these agents can be used to diagnose, treat, or both diagnose and treat cancer and inflammatory or infectious diseases [2]. Gamma- and positron-emitting radionuclides support diagnostic imaging via single-photon emission computed tomography (SPECT) and positron emission tomography (PET), with more than 80% of medical radionuclides employed for imaging purposes [3]. In therapeutic applications, alpha and beta particles, as well as Auger electrons, induce cytotoxic effects, generating reactive oxygen species, inducing DNA damage, and impairing DNA repair mechanisms [3].

When a single agent offers both diagnostic and therapeutic functions, the concept of theranostics applies. Theranostic radiopharmaceuticals either emit both therapeutic and imaging radiation (e.g., beta and gamma) or consist of separate agents that target the same biomolecule while emitting different types of radiation [4,5]. This approach holds promise for personalized medicine. Despite their potential, conventional radiopharmaceuticals face critical limitations. Challenges include radionuclide purity, handling, stability, radiolysis, drug interactions, extravasation, and toxicity [6,7]. Systemic distribution may lead to off-target accumulation, particularly in the kidneys, risking nephrotoxicity [8,9]. Moreover, traditional molecular delivery systems transport only limited radionuclide loads, requiring high doses that may exacerbate adverse effects.

In this context, the development of nanoradiopharmaceuticals, nanoscale systems radiolabeled for diagnostic and/or therapeutic purposes, emerges not as a redundant innovation but as a critical response to the shortcomings of conventional agents. Nanoparticles offer a unique combination of physicochemical properties: high surface-to-volume ratios, tunable composition, and versatile surface chemistry. These features enable enhanced radionuclide loading through multivalent coordination or encapsulation, as well as precise control over pharmacokinetics and biodistribution. Importantly, their size and surface functionality promote both passive tumor accumulation via the enhanced permeability and retention (EPR) effect and active targeting through ligand–receptor interactions. These mechanisms can significantly increase target-site retention and reduce background signal, resulting in improved imaging sensitivity and therapeutic efficacy [10,11]. Comparative preclinical studies have demonstrated that radiolabeled nanoparticles often achieve higher tumor-to-background ratios and more prolonged intratumoral retention than small-molecule radiopharmaceuticals, even when using the same radionuclide. Furthermore, the modularity of nanomaterials allows for the integration of dual-modality imaging, controlled drug release, and enzyme-responsive designs, functionalities that are difficult to achieve with traditional systems [12].

Emerging molecular strategies highlight the potential for integrating advanced targeting mechanisms into nanoradiopharmaceuticals to further personalize medicine. For example, radiolabeled fluoroquinolones, such as those labeled with ^99m^Tc, have been developed to selectively bind bacterial receptors for imaging infections, demonstrating high specificity in preclinical models [13]. Similarly, radiolabeled neuropeptides engineered with cathepsin B cleavage sites have shown improved tumor-to-nontarget tissue ratios by modulating intracellular trafficking, allowing the controlled release of radiometabolites [14]. These approaches, while currently applied to small molecules or peptides, could be adapted to nanoparticle platforms, where surface functionalization with specific ligands or enzyme-cleavable sequences could enhance targeting precision and optimize biodistribution. By incorporating such molecular designs into nanomaterials, nanoradiopharmaceuticals could achieve greater control over radionuclide delivery and metabolism, paving the way for tailored diagnostics and therapies that align with the principles of personalized medicine.

**Figure 1:**
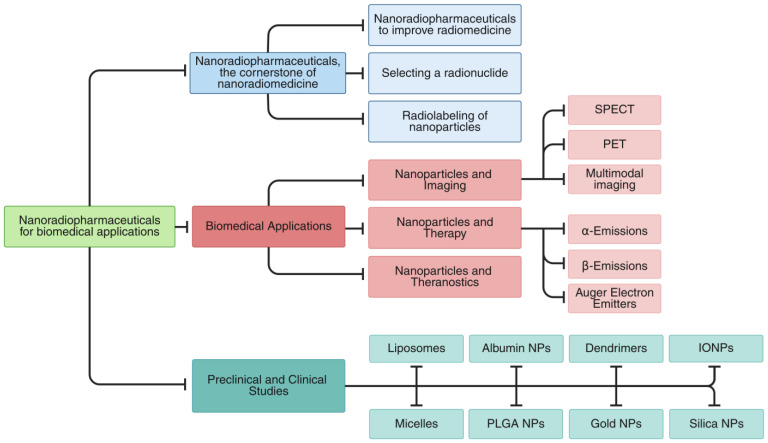
Scheme of the scope and key sections covered in this review. Created with BioRender.com. Created in BioRender. Núñez-Salinas, A. (2025) https://BioRender.com/ku33foj (accessed on 30 May 2025).

In this review, we present an integrated overview of nanoradiopharmaceuticals, covering both the fundamental principles and the latest advancements in the field. The review is structured into several key sections (Figure 1).

This review begins by examining the foundational aspects of nanoradiopharmaceutical design, including strategies to advance nanomedicine, criteria for radionuclide selection, and nanoparticle radiolabeling methodologies (Section 2). We then examine key biomedical applications, covering nanoparticle-based imaging (SPECT, PET, multimodal), therapeutic approaches (β-, α-, Auger electron emitters), and theranostic constructs that unify diagnostic and therapeutic functions (Section 3). Section 4 provides a critical assessment of both preclinical and clinical investigations involving radiolabeled nanoparticles across a range of carriers (liposomes, micelles, albumin, PLGA, dendrimers, gold, iron oxide, and silica). We then synthesize these findings to discuss current limitations, translational challenges, and opportunities for future innovation (Section 5). Finally, we distill the major insights and outline prospects for advancing the field (Section 6).

Each section delves with focused insight into key facets of nanoradiopharmaceuticals, from their design and development to their applications in imaging and therapy, supported with both preclinical and clinical studies. This structured progression guides readers through the current landscape of the field while illuminating future directions in nanomedicine.

## 2. Nanoradiopharmaceuticals, the Cornerstone of Radionanomedicine

### 2.1. Nanoradiopharmaceuticals to Improve Nanomedicine

The convergence of nanotechnology and nuclear medicine has given rise to the field of radionanomedicine, which leverages the unique properties of nanomaterials to enhance the performance of radiopharmaceuticals. The early use of radionuclide-labeled nanoparticles focused on tracing biodistribution and pharmacokinetics. Today, nanoradiopharmaceuticals are being developed for a broad spectrum of clinical applications, ranging from imaging to therapy and theranostics.

At the core of radionanomedicine is the integration of radionuclides onto or within nanomaterials, enabling the use of trace amounts for in vivo imaging and therapy. Radionanomedicine enhances the specificity, sensitivity, and multifunctionality of radiopharmaceuticals, facilitating their use in several key areas:

- Diagnostic imaging: radiolabeled nanoparticles can improve imaging quality in PET and SPECT by enhancing uptake and retention in target tissues [15].
- Targeted therapy: functionalized nanoparticles can deliver therapeutic radiation selectively to tumors, minimizing systemic side effects [16,17].
- Theranostics: dual-function nanoparticles combine diagnostics and therapy in a single agent, allowing the real-time monitoring of a treatment response [18].
- Disease monitoring: the in vivo tracking of nanoparticle distribution supports personalized medicine by evaluating therapeutic efficacy and disease progression [19].
- Early detection: the high sensitivity of radiolabeled systems facilitates early-stage diagnosis, which is critical for improved clinical outcomes [20].

These applications are underpinned by several physicochemical and biological properties of nanoparticles: high sensitivity and payload [21], targeting specificity [22,23,24], controlled size and shape [21,25], surface charge and biocompatibility [2,26], multifunctionality [27,28], in vivo tracking and radiochemical stability [24,29], and therapeutic isotope diversity [29].

Currently, several radiolabeled nanoparticles are in preclinical development, with some progressing to clinical trials [30,31,32,33].

### 2.2. Selecting a Radionuclide

Radionuclides used in medicine are primarily obtained from three sources: nuclear reactors, particle accelerators, and radionuclide generators [34,35,36]. Among these, low-energy cyclotrons (10–30 MeV), have become increasingly relevant due to their ability to produce a broad spectrum of radionuclides—including positron and gamma emitters—directly within clinical facilities. Radionuclide generators offer further advantages by enabling on-site production of short-lived isotopes without the need for centralized infrastructure.

Selecting a suitable radionuclide for diagnostic or therapeutic applications requires a careful consideration of both physical and biochemical parameters. Key physical characteristics include half-life, decay mode, and type of emission, which influence not only tissue penetration and radiation dose distribution (crucial in therapy) but also image resolution, detection efficiency, and background signal (essential in diagnostic imaging) [3]. Biochemical factors, such as in vivo stability, toxicity, and metabolic behavior, must also be assessed, alongside clinical parameters like tumor size, location, vascularization, and receptor expression [37]. The method of radionuclide incorporation into nanoparticles—via direct conjugation, chelation, or encapsulation—critically affects stability and biodistribution [3,38].

### 2.3. Radiolabeling of Nanoparticles

**Figure 2:**
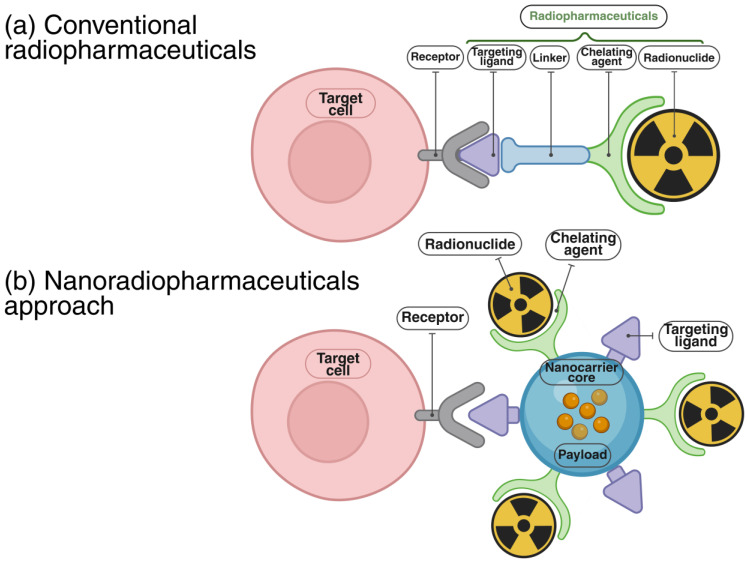
Schematic representation of radiolabeling. (**a**) Conventional radiopharmaceuticals and (**b**) nanoradiopharmaceuticals approach. Created with BioRender.com. Created in BioRender. Núñez-Salinas, A. (2025) https://BioRender.com/mob2xx0 (accessed on 30 May 2025).

Radiolabeling strategies for nanoparticles vary, depending on the physicochemical properties of both the nanomaterial and the radionuclide [9,39,40]. A key consideration is the in vivo stability of the radiolabel, which refers to the preservation of the radiochemical integrity and strong binding of the radionuclide to the nanoparticle in biological environments, preventing its premature release. In parallel, the physical half-life of the selected radionuclide should be compatible with the circulation and biodistribution profile of the nanoparticle to ensure effective imaging or therapy. Similar to conventional radiopharmaceuticals, radiolabeled nanoparticles typically consist of four components: a nanocarrier core, a targeting ligand, a chelating agent, and a radionuclide (Figure 2).

Radiolabeling methods are commonly categorized as extrinsic or intrinsic [41]. Extrinsic radiolabeling entails the chemical conjugation of radionuclides to the nanoparticle surface, typically using bifunctional chelators capable of coordinating both the radionuclide and the nanoparticle [42,43]. Common chelators include diethylenetriaminepentaacetic acid (DTPA), 1,4,7,10-tetraazacyclododecane-1,4,7,10-tetraacetic acid (DOTA), and 1,4,7-triazacyclononane-1,4,7-triacetic acid (NOTA), each offering strong bonding affinity and broad applicability across various radiometals [44,45,46]. This approach is widely adopted due to its simplicity and commercial availability. However, the potential in vivo release of the radiometal can lead to off-target accumulation, thereby diminishing imaging contrast and increasing radiation exposure to healthy tissues.

Intrinsic radiolabeling, by contrast, incorporates the radionuclide directly into the nanoparticle matrix during or after synthesis, resulting in enhanced in vivo stability [21,47]. This integration minimizes the risk of radiometal detachment under physiological conditions, thereby improving imaging fidelity and reducing non-specific tissue accumulation. Four principal intrinsic strategies have been delineated:

1. Hot-plus-cold precursors: Radioactive and non-radioactive precursors are co-synthesized, with their ratio defining the specific activity. For example, ^64^Cu-labeled copper sulfide nanoparticles have been synthesized using this method [48].
2. Specific trapping: Some nanomaterials exhibit selective affinity for certain radionuclides, allowing spontaneous incorporation without chemical modification. Chimeric nanoparticles capable of trapping ^64^Cu serve as a representative example [49].
3. Cation exchange: Radiometals are introduced via ion exchange with metal ions present on the nanoparticle surface. A notable case is the synthesis of NaLuF_4_:Yb,Tm@NaGdF_4_ core–shell nanoparticles doped with ^153^Sm^3+^, enabling multimodal imaging [50].
4. Proton beam activation: Nanoparticles composed of stable isotopes are directly irradiated with a proton beam to induce nuclear reactions, generating radioactive isotopes in situ. This has been applied to the radiolabeling of nanoparticles with ^18^F [51].

Intrinsic methods reduce the risk of radionuclide dissociation in vivo, enhancing target specificity and safety. The choice between extrinsic and intrinsic radiolabeling depends on multiple factors, including radionuclide chemistry, nanoparticle composition, required specific activity, and production feasibility. Ultimately, optimizing radiolabeling strategies is crucial for developing safe, effective, and clinically translatable nanoradiopharmaceuticals.

## 3. Biomedical Applications

Radionuclide-labeled nanomaterials have gained significant attention in recent years, giving rise to the emerging field of radionanomedicine, which bridges nuclear medicine and nanotechnology [12,52]. Nanoparticles composed of high-atomic-number elements enhance radiological contrast in imaging modalities such as computed tomography (CT) and magnetic resonance imaging (MRI) [12]. Multifunctional platforms can be engineered to both detect and treat tumors [53,54,55], and they may also act as radiosensitizers to potentiate radiation therapy [3]. In addition, radioisotope incorporation improves selective accumulation, the signal-to-noise ratio in imaging, and therapeutic efficacy [54]. Several of these applications are discussed below.

### 3.1. Nanoparticles and Imaging

Medical imaging is divided into anatomical techniques, MRI and CT—which offer high-resolution structural visualization (MRI excels in soft-tissue contrast; CT in spatial resolution and deep penetration)—and functional modalities, optical imaging and nuclear methods (PET, SPECT)—which enable real-time assessment of physiological and molecular events [56,57,58]. Each modality presents distinct trade-offs: optical imaging provides high sensitivity and rapid acquisition but is limited in depth and spatial resolution; MRI and CT deliver detailed, whole-body anatomical images but lack molecular sensitivity, and CT involves ionizing radiation; PET and SPECT offer quantitative, highly sensitive molecular imaging with effective tissue penetration, albeit with lower spatial resolution, radiation exposure, and, in the case of PET, often requiring a cyclotron for radionuclide production.

Multimodal imaging techniques, such as PET/MRI and SPECT/CT, integrate the molecular sensitivity of nuclear methods with the anatomical precision of MRI or CT, thereby addressing the limitations of single-modality imaging. Central to this evolution, molecular imaging enables visualization of biochemical processes preceding structural alterations, facilitating early diagnosis, and personalized therapy [59,60,61]. Nanoparticles further enhance these capabilities through their tunable surfaces and high surface-to-volume ratios, accommodating multiple imaging moieties (e.g., radionuclides, fluorophores, targeting ligands) that improve radiostability, payload capacity, and target specificity [43,55,62]. Radiolabeled nanoparticles enable in vivo tracking, quantitative imaging, and theranostic applications, particularly in oncology. Compared to conventional radiotracers, they exhibit superior stability, higher isotope payloads, and enhanced target precision, thereby minimizing off-target effects [63,64]. While traditional imaging modalities (e.g., radiography, ultrasound, MRI) typically detect disease only after morphological changes occur, PET and SPECT excel in identifying early molecular alterations, complementing anatomical imaging for accurate disease localization and characterization.

Consequently, the convergence of molecular imaging, nanotechnology, and radiopharmacy is reshaping the diagnostic landscape. Radiolabeled nanoparticles are paving the way for highly sensitive, specific, and personalized imaging approaches that not only detect disease earlier but also monitor therapeutic efficacy and guide treatment decisions. The following sections highlight recent advancements and biomedical applications of radiolabeled nanoparticles in molecular imaging, with a particular emphasis on multimodal and theranostic approaches.

#### 3.1.1. Single-Photon Emission Computed Tomography (SPECT)

SPECT is a nuclear imaging modality that detects gamma photons emitted via radiotracers accumulated in target tissues. A key advantage of SPECT lies in its extended imaging window, made possible through the relatively long half-lives of single-photon-emitting radionuclides. This feature enables the real-time monitoring of biological processes over several hours or even days post-administration [65]. Commonly used radionuclides include technetium-99m (^99m^Tc, t_1_/_2_: 6.02 h), gallium-67 (^67^Ga, t_1_/_2_: 3.26 d), indium-111 (^111^In, t_1_/_2_: 2.8 d), and iodine-131 (^131^I, t_1_/_2_: 8.02 d). Notably, ^111^In and ^131^I also emit Auger and β^−^ particles, respectively, enabling therapeutic applications.

Nanoparticles labeled with SPECT radionuclides have demonstrated significant potential for targeted imaging. For instance, gold nanoparticles functionalized with gallic acid and labeled with ^99m^Tc showed efficient tumor accumulation following both intravenous and intratumoral administration in animal models [66]. The optimization of the radiolabeling process involved parameters such as reducing agent concentration, pH, and incubation time. In a separate study, ^99m^Tc-labeled copper oxide nanoparticles synthesized using *Aspergillus flavus* achieved a high radiolabeling yield (~93%) and exhibited selective tumor accumulation, as well as antimicrobial activity and cancer-specific cytotoxicity [67].

These findings underscore the potential of radiolabeled nanoparticles as dual diagnostic–therapeutic agents, thereby expanding the utility of SPECT within the field of nanomedicine.

#### 3.1.2. Positron Emission Tomography (PET)

PET is an advanced molecular imaging technique that relies on the detection of coincident gamma photons generated via positron–electron annihilation events. Unlike SPECT, which detects single gamma photons, PET captures pairs of 511 keV photons emitted in opposite directions, enabling high-precision three-dimensional localization of radiotracers within the body [65]. PET offers superior sensitivity and quantitative accuracy, making it particularly well suited for dynamic and functional imaging applications [68]. Common PET radioisotopes include ^18^F (t_1_/_2_: 109.8 min), ^64^Cu (t_1_/_2_: 12.7 h), ^68^Ga (t_1_/_2_: 67.7 min), ^89^Zr (t_1_/_2_: 78.4 h), and ^124^I (t_1_/_2_: 4.18 d), all of which emit positrons suitable for high-resolution imaging. For instance, Unak et al. developed ^18^F-labeled gold nanoparticles conjugated with anti-metadherin antibodies and 2-deoxy-2-fluoro-D-glucose (^18^FDG), demonstrating specific uptake in breast cancer cells [15]. Similarly, Hajiramezanali et al. synthesized ^68^Ga-labeled, trimethyl chitosan-coated superparamagnetic nanoparticles conjugated with bombesin, exhibiting high affinity for T-47D cells and tumor-specific accumulation in vivo [69]. These studies underscore the potential of radiolabeled nanoparticles in PET imaging for targeted cancer diagnostics and theranostic applications.

#### 3.1.3. Multimodal Imaging

Recent advances in hybrid imaging have driven the rapid development of multimodal probes, effectively addressing the inherent limitations of single-modality techniques [60]. Hybrid systems such as PET/CT and SPECT/CT are well established in clinical practice, while PET/MRI and SPECT/MRI are gaining traction in both preclinical and clinical research due to their ability to provide high-resolution soft-tissue contrast with reduced radiation exposure [70]. The integration of nanotechnology into multimodal imaging has further propelled the field, enabling the creation of functionalized nanoparticles designed to target specific biomarkers and enhance signal contrast across imaging modalities.

Among targeted nanosystems, atrial natriuretic peptide (ANP) has attracted interest for its anticancer activity mediated through natriuretic peptide receptors NPRA and NPRC. Pressly et al. developed ^64^Cu-labeled ANP-conjugated nanoparticles (^64^Cu-CANF-Comb) for the PET imaging of NPRC in prostate cancer [71], demonstrating favorable pharmacokinetics, including prolonged circulation time and reduced hepatic uptake. Similarly, ^68^Ga-labeled carbon nanoparticles have been proposed as an alternative to Technegas^®^ for pulmonary ventilation imaging. Blanc-Béguin et al. reported that these particles exhibit physicochemical properties, such as particle size and density, comparable to Technegas^®^, and show promising performance in PET/CT-based lung ventilation imaging [72]. Also, Yang et al. investigated the pharmacokinetics and radiosensitizing capabilities of platinum-based nanostructures using PET/CT imaging. Of the two systems evaluated, the flower-like PtNF-PA architecture displayed superior tumor accumulation, sustained retention, and enhanced radiotherapeutic efficacy in a melanoma model, underscoring the importance of nanoparticle morphology in therapeutic outcomes [73].

**Figure 3:**
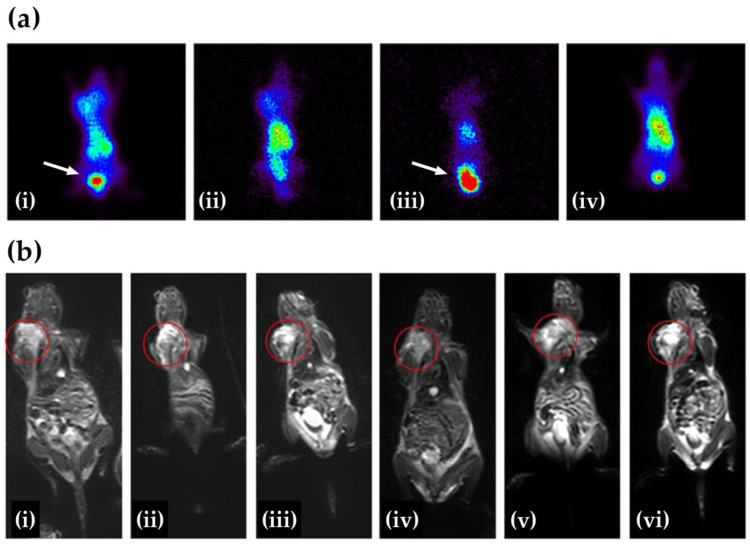
Tumor-specific accumulation of VEGF-targeting IONPs visualized with gamma-ray and MR imaging. (**a**) Significant tumor accumulation of the radiotracer at 1 h (**i**), and 24 h post-injection (**ii**), no tumor uptake when blocked with excess bevacizumab (**iii**), and minimal tumor uptake with the non-targeted nanoconstruct (**iv**). (**b**) High tumor signal intensity in T2-weighted images before nanoparticle administration (**i**–**iii**), decreased signal intensity after administration of the targeted nanoconstruct, indicating specific uptake (**iv**), slight signal drop with the non-targeted nanoconstruct (**v**), and no change in control mice (**vi**). The white arrow indicates the accumulation. Reproduced with permission [76].

The PET/MRI platform synergistically combines the high anatomical resolution of MRI with the molecular sensitivity of PET. To support this dual functionality, various magnetic materials, such as manganese (Mn), gadolinium (Gd), copper (Cu), zirconium (Zr), and iron oxide, have been incorporated into multifunctional nano-based systems. Notably, Schütz et al. synthesized ^18^F-labeled iron oxide nanoparticles functionalized with catechol, folic acid, and dopamine, demonstrating their suitability for dual PET/MRI imaging in both in vitro and in vivo settings [74]. In addition, intrinsically magnetic nanoparticles labeled with ^64^Cu or ^68^Ga have shown efficacy in PET/MRI applications [75]. Exampling a complementary approach, Tsoukalas et al. developed superparamagnetic iron oxide nanoparticles (SPIONs) coated with dimercaptosuccinic acid (DMSA) and labeled with ^99m^Tc for SPECT/MRI imaging. These probes enabled the in vivo visualization of angiogenesis, providing both functional and anatomical insights within a single session (Figure 3) [76].

Optical imaging modalities, including near-infrared fluorescence (NIRF) and photoacoustic imaging (PA), have been integrated with nuclear imaging platforms to address the limitations of each technique [77,78,79]. In one such example, Harmsen et al. developed PET/NIRF silica nanoparticles for longitudinal tracking of chimeric antigen receptor (CAR) T cells. These nanoparticles exhibited stable intracellular retention and allowed the non-invasive in vivo monitoring of T cell distribution for up to one week [80].

An additional emerging modality is Cerenkov radiation energy transfer (CRET), which exploits the light emitted when charged particles exceed the phase velocity of light in a dielectric medium [81]. Although limited due to shallow tissue penetration, Cerenkov radiation’s short-wavelength emission can be absorbed and re-emitted in the near-infrared (NIR) range by certain nanomaterials. Carbon dots, quantum dots, gold nanoparticles, and upconversion nanoparticles have been shown to convert Cerenkov radiation into NIR light, thereby enhancing imaging sensitivity and tissue penetration [82,83,84].

Fluorescence imaging, widely employed for subcellular visualization, offers high resolution and cost-effectiveness, but it is limited due to shallow tissue penetration. Its integration with PET or SPECT helps overcome these limitations, particularly in preclinical research. Fluorescent probes, such as quantum dots, gold nanoparticles, and organic dyes, have been conjugated with radioisotopes, including ^18^F, ^124^I, ^125^I, and ^64^Cu, to develop PET/fluorescence or SPECT/fluorescence bimodal agents [85,86]. These hybrid probes enable highly sensitive, high-resolution imaging at both the macroscopic and microscopic levels.

These examples highlight the transformative potential of nanoparticle-based multimodal imaging platforms. By integrating complementary imaging modalities into a single nanoconstruct, these systems enable improved anatomical localization, precise molecular targeting, and real-time therapy monitoring. As advances in imaging technologies and nanomaterial engineering progress, the development of multifunctional, clinically translatable probes is poised to redefine diagnosis, treatment planning, and outcome prediction in the emerging landscape of nanomedicine.

### 3.2. Nanoparticles and Therapy

Beyond diagnostic applications, nanomaterials have been widely investigated for therapeutic purposes, particularly in drug delivery, photodynamic therapy, photothermal therapy, and radiotherapy. Their ability to encapsulate therapeutic agents and selectively accumulate in pathological tissues renders them ideal candidates for targeted treatment strategies. In radiotherapy, nanoparticles can serve as carriers for therapeutic radionuclides, enabling the localized emission of ionizing radiation at the tumor site while minimizing damage to surrounding healthy tissues. The therapeutic efficacy of ionizing radiation primarily stems from DNA damage, especially double-strand breaks, and indirect oxidative damage mediated via reactive oxygen species (ROS). For instance, radiation-induced water radiolysis generates free radicals, such as hydroxyl (^●^OH), hydrogen (H^●^), and superoxide (O_2_^●−^), which in turn damage nucleic acids, proteins, lipids, and other cellular components [87].

**Figure 4:**
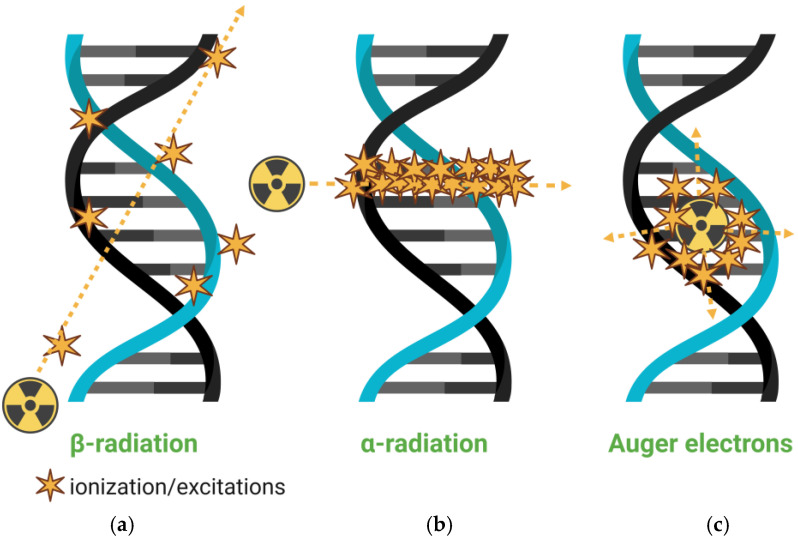
Illustration of therapeutic radionuclide emissions. (**a**) Beta, (**b**) alpha, and (**c**) Auger electron emissions. Created with BioRender.com. Created in BioRender. Núñez-Salinas, A. (2025) https://BioRender.com/gpteyg0 (accessed on 30 May 2025).

A major challenge in radionuclide therapy is achieving high tumor specificity to reduce off-target toxicity. Nanotechnology presents a promising solution through the design of nanocarriers with enhanced tumor accumulation and prolonged retention, typically mediated via the enhanced permeability and retention (EPR) effect or active targeting strategies [9]. Therapeutic radionuclides used in nanoparticle-based radiotherapy include alpha, beta, and Auger electron emitters (Figure 4), each offering distinct radiobiological advantages depending on tumor characteristics and clinical objectives. Table 1 summarizes their characteristics.

### 3.3. Nanoparticles and Theranostics

As previously discussed, nanoparticles serve a pivotal role in this field by enabling the co-delivery of imaging agents, therapeutic radionuclides, and targeting ligands within a unified construct [91,92]. The selection of a radionuclide is dictated by its emission characteristics—gamma or positron emitters are employed for imaging, whereas beta or alpha emitters are used for therapeutic purposes. Theranostic radionuclide pairs, such as ^99m^Tc/^188^Re, ^68^Ga/^177^Lu, ^68^Ga/^225^Ac, and ^89^Zr/^177^Lu [93,94,95] allow for sequential diagnostic and therapeutic applications through chemically analogous radioconjugates. Alternatively, isotopes such as ^131^I, ^177^Lu, and ^212^Pb, are natural theranostic radioisotopes since they emit gamma radiation and beta or alpha particles at the same time [70]. This dual functionality facilitates real-time tracking, individualized dosimetry, and enhanced therapeutic precision with reduced systemic toxicity [54,96]. A number of recent studies have demonstrated the versatility of this approach using diverse nanoparticle systems. For example, Gibbens-Bandala et al. developed a multifunctional dendrimer-based nanoplatform (DOTA-DN-BN) designed to deliver triple-modal therapy for breast cancer [97]. This multifunctional construct integrated the following: (1) ^177^Lu radiotherapy via DOTA chelation, (2) paclitaxel chemotherapy, and (3) active targeting through a bombesin-derived peptide selective for gastrin-releasing peptide receptors (GRPr), which are frequently overexpressed in breast cancer. The nanosystem demonstrated high physicochemical stability, controlled drug release, and pronounced cytotoxicity in GRPr-positive cell lines. In vivo biodistribution studies confirmed preferential tumor accumulation and favorable dosimetric profiles. In a related study, Zhang et al. reported the development of ^131^I-labeled copper sulfide nanoparticles coated with bovine serum albumin (BSA@CuS) for combined radio-photothermal therapy targeting anaplastic thyroid cancer [98]. Following intratumoral injection and 808 nm laser irradiation, the BSA@CuS nanoparticles induced synergistic cytotoxic effects via both thermal ablation and radiotherapeutic mechanisms. SPECT/CT imaging verified tumor-localized nanoparticle accumulation, and in vivo evaluations revealed superior therapeutic efficacy compared to either modality alone.

Collectively, these studies demonstrate the multifaceted advantages of nanosystems in theranostic applications, including precise cellular targeting, controlled biodistribution, and the integration of multimodal imaging and therapeutic functionalities. Such features contribute to enhanced treatment efficacy and reduced systemic toxicity, positioning nanoscale platforms as powerful enablers of personalized medicine.

## 4. Preclinical and Clinical Studies of Radiolabeled Nanoparticles

The field of cancer nanomedicine has witnessed substantial advancements in recent years, marked by the clinical approval of several nanoparticle-based therapeutics and a growing number of candidates entering late-stage clinical trials [99]. Within this expanding landscape, radiolabeled nanoparticles have emerged as particularly promising platforms, offering integrated multifunctionality that bridges diagnostic imaging and targeted therapy. Preclinical investigations consistently highlight their favorable pharmacokinetic behavior, superior tumor accumulation, and adaptability for multimodal applications. Collectively, these attributes position radiolabeled nanoparticles at the forefront of precision oncology, with the potential to refine therapeutic efficacy and enable patient-specific strategies.

Despite encouraging preclinical results, the clinical translation of radiolabeled nanosystems remains constrained. To date, most human studies have focused on radiolabeled liposomal formulations, reflecting both the technical feasibility and the relative maturity of liposome-based delivery platforms. Early clinical trials with these systems have established a proof of concept for tumor-targeted delivery and image-guided therapy, while simultaneously highlighting key translational challenges. Critical barriers include nanoparticle stability, radiolabel retention, immunogenic responses, and the complexity associated with large-scale manufacturing. Moreover, regulatory hurdles and the absence of standardized characterization protocols underscore the need to align preclinical advances with clinical and regulatory frameworks. The limited number of ongoing clinical trials further emphasizes the urgency of developing refined targeting strategies, implementing harmonized quality control measures, and conducting long-term safety assessments. These efforts are essential to bridge the gap between laboratory success and clinical impact, ensuring that the full potential of radiolabeled nanomedicine is realized for patient benefit. In this context, we review preclinical and clinical investigations of eight radiolabeled nanosystems, with the aim of elucidating their biomedical applications and prospective impact in clinical settings.

### 4.1. Liposomes

**Figure 5:**
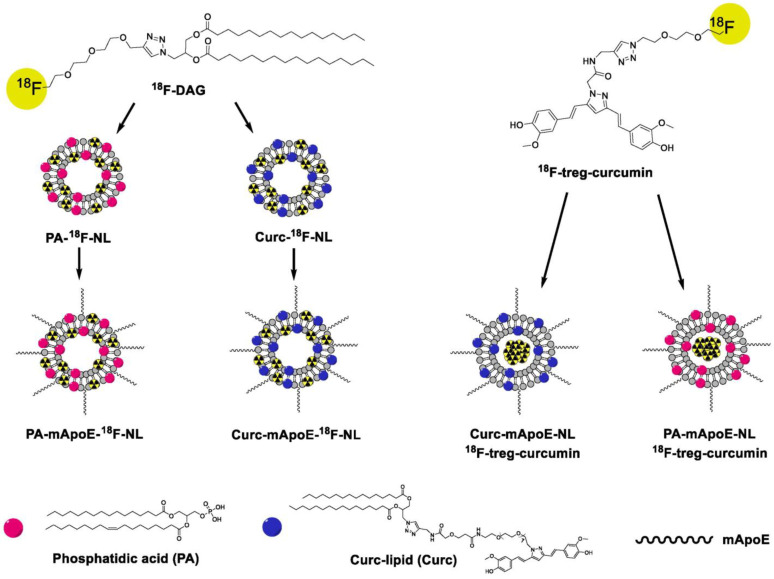
Illustration of ^18^F-labeled nanoliposomes (^18^F-NL) conjugated with monomeric ApoE peptide. The synthesis sequence involved initial ^18^F-labeling, followed by the preparation of phosphatidic acid (PA)- or lipidic treg-curcumin (Curc)-functionalized liposomes, which were then further functionalized with mApoE to produce ^18^F-NLs. Reproduced with permission [101].

Liposomal systems have emerged as one of the most established and adaptable nanocarriers in biomedical science, particularly within drug delivery and radiopharmaceutical applications. Since the landmark FDA approval of liposomal doxorubicin (Doxil^®^) in 1995, the clinical translation of liposome-based therapeutics has steadily progressed, with numerous formulations advancing through clinical evaluation and entering routine medical use. Owing to their structural duality, liposomes can encapsulate hydrophilic radionuclides within their aqueous core or incorporate lipophilic radiolabeled compounds into the lipid bilayer, enabling multifunctional applications in both nuclear imaging and targeted radiotherapy. Preclinical studies have provided compelling evidence supporting the utility of radiolabeled liposomes across a spectrum of diagnostic and therapeutic scenarios. Recently, Alkandari et al. investigated cationic liposomes encapsulating ^99m^Tc-sestamibi, formulated with DPPC, cholesterol, and stearyl amine to yield unilamellar vesicles. The phospholipid headgroups (DPPC) and stearyl amine provide amino functionalities that coordinate ^99m^Tc. No active ligands are grafted; instead, passive targeting relies on liposome size and charge to influence biodistribution. In rabbit models, these liposomes enhanced myocardial uptake, accelerated clearance, and reduced hepatic accumulation compared to the free tracer, demonstrating the pharmacokinetic advantages of liposomal encapsulation for cardiac imaging [100]. In the context of neurodegenerative disease, Rokka et al. developed ^18^F-labeled nanoliposomes (Figure 5) [101]. The targeting of Alzheimer’s-related β-amyloid (Aβ) plaques was pursued through surface functionalization with a monomeric ApoE peptide (mApoE), which binds to low-density lipoprotein receptors (LDLr) on brain endothelial cells, enhancing transport across the blood–brain barrier (BBB). These theranostic agents represent a major advancement in imaging CNS disorders using liposomes, enabling improved diagnosis and treatment.

Further insights into interpatient variability were provided in the study by Lee et al., in which ^64^Cu-MM-302 was administered to 19 patients with HER2-positive breast cancer [102]. This PEGylated liposomal formulation (spherical vesicles) encapsulates doxorubicin and is functionalized for HER2 targeting. ^64^Cu was incorporated using a bifunctional chelator, which forms a stable complex with the radionuclide and anchors it to the liposome surface during the post-labeling process. PET/CT imaging revealed a striking 35-fold variation in tumor uptake among patients, which correlated with therapeutic response. These findings highlighted the potential of radiolabeled liposomes not only as vehicles for targeted delivery but also as predictive biomarkers, reinforcing the need for personalized nanomedicine strategies in oncologic care.

### 4.2. Micelles

Micellar systems, particularly those based on amphiphilic block copolymers, have garnered considerable interest in nanomedicine due to their structural adaptability, efficient drug-loading capabilities, and ease of functionalization and radiolabeling. These nanoscale assemblies feature a core–shell architecture, wherein the hydrophobic core encapsulates poorly water-soluble drugs or lipophilic radionuclides, while the hydrophilic corona confers aqueous stability and extends systemic circulation. With typical sizes ranging from 10 to 100 nm, micelles preferentially accumulate in tumor tissues via the enhanced permeability and retention (EPR) effect, rendering them highly promising platforms for imaging and therapeutic applications in oncology and other disease contexts [103,104].

**Figure 6:**
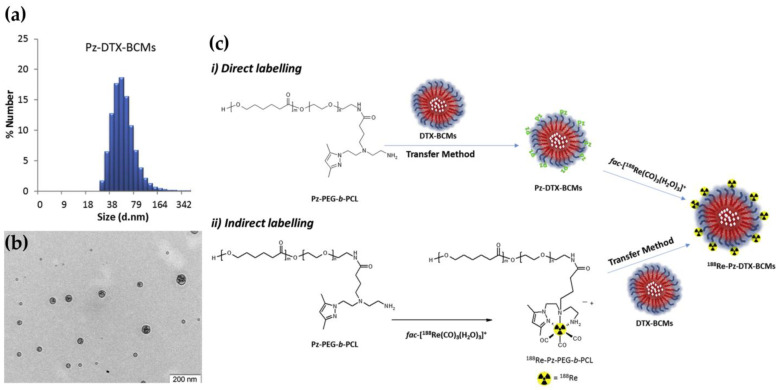
Schematic illustration of the preparation of docetaxel-loaded block copolymer micelles labeled with ^188^Re. (**a**) DLS histogram and (**b**) TEM image for the functionalized docetaxel-loaded micelles with a pyrazolyl-diamine chelating unit; (**c**) synthesis scheme. Reproduced with permission [105].

Micelles provide a modular platform amenable to both passive and active targeting strategies. The incorporation of targeting ligands, such as antibodies, aptamers, peptides, or small molecules, expands their specificity and promotes cellular uptake in disease-relevant tissues. In a preclinical study, Ribeiro et al. demonstrated the co-delivery of docetaxel (DTX) and the beta-emitter ^188^Re using block copolymer micelles, thereby establishing a synergistic chemoradiotherapy platform [105]. These micelles exhibit spherical morphology, with a hydrophobic polycaprolactone (PCL) core and a hydrophilic PEG corona. The micelles are functionalized with pyrazolyl-diamine ligands that react with the *fac*-[^188^Re(CO)_3_(H_2_O)_3_]^+^ complex, anchoring the ^188^Re via coordination to nitrogen donor atoms on the ligand (Figure 6). The micelles are designed for passive tumor targeting via the enhanced permeability and retention (EPR) effect. The formulation exhibited prolonged systemic circulation, high colloidal stability, and improved tumor targeting. Notably, direct radiolabeling methods achieved high radiochemical purity and stability, reinforcing the promise of micellar systems for combinatorial therapeutic applications.

Micelles have also been investigated in the context of liquid brachytherapy (LBT), a minimally invasive alternative to conventional brachytherapy that employs injectable radiolabeled formulations. In a study by Wang et al. (2020), cationic micelles and liposomes were radiolabeled with ^64^Cu and ^177^Lu using DOTA-triarginine-lipid conjugates (D3R-C16 and D3R-C18) [106]. Among the tested formulations, D3R-C18 micelles exhibited radiochemical purity exceeding 99% and favorable in vivo biodistribution, with more homogeneous intratumoral radioactivity compared to liposomes, suggesting superior performance in localized radiotherapy. The triarginine moiety provides a cationic character, favoring electrostatic interactions with negatively charged cell membranes, allowing membrane insertion and intercellular diffusion within tumor tissue. This mechanism promotes homogeneous intratumoral distribution and supports its application in liquid brachytherapy, especially after direct intratumoral injection.

Overcoming central nervous system (CNS) barriers remains a major challenge in drug delivery due to the restrictive nature of the blood–brain barrier (BBB). Upadhaya et al. evaluated the intranasal administration of methotrexate (MTX)-loaded spherical polymeric micelles radiolabeled with ^99m^Tc for the treatment of glioblastoma [107]. Here, the MTX-TEPA conjugate is radiolabeled using ^99m^Tc in the presence of stannous chloride (SnCl_2_) as a reducing agent. The TEPA moiety acts as a bifunctional chelator, providing nitrogen donor atoms for coordination with ^99m^Tc. By leveraging folate receptor overexpression in glioma cells and exploiting the nasal-brain transport route, the micellar formulation achieved enhanced brain accumulation and demonstrated threefold increased cytotoxicity in U87-MG cells compared to MTX in solution. SPECT imaging corroborated CNS delivery. Targeting is achieved via mucoadhesion and nose-to-brain transport. The surfactant Solutol HS15 contributes to mucoadhesion, allowing longer residence time in the nasal cavity, while the nanoscale size facilitates paracellular absorption and neuronal transport. To further improve tumor selectivity, Salgueiro et al. (2024) designed micelles functionalized with glucose- and bevacizumab, targeting overexpressed glucose transporters and angiogenic markers in tumors [108]. Radiolabeling with ^99m^Tc is performed via a direct labeling method using SnCl_2_ as a reducing agent. The labeling targets accessible functional groups in Soluplus^®®^ polymer chains, likely hydroxyl or ether groups. This radiolabeling yielded high radiochemical purity (>95%) and efficient tumor localization in breast and colon cancer models. Importantly, ligand conjugation did not alter particle size (~100 nm) or zeta potential, preserving colloidal stability and systemic compatibility.

### 4.3. Albumin Nanoparticles

Albumin, the most abundant protein in blood plasma, is widely recognized for its biocompatibility and has been extensively investigated as a nanocarrier in cancer therapy. Its versatile surface chemistry, conferred by reactive thiol, amine, and carboxyl functional groups, enables the conjugation of therapeutic agents, imaging probes, and targeting ligands, facilitating the development of multifunctional albumin-based nanoparticles [109,110]. Notably, the U.S. Food and Drug Administration has recognized albumin as a clinically approved drug delivery vehicle, particularly in oncological applications.

In oncology, thyroid cancer stands as the most prevalent endocrine malignancy, with rising incidence rates often attributed to overdiagnosis. Standard treatment typically involves total thyroidectomy, lymph node resection, and radioactive iodine therapy using ^131^I [111]. As an innovative approach, Zhang et al. developed BSA nanoparticles conjugated with iodine-131 and copper sulfide (^131^I-BSA@CuS) for combined radiotherapy and photothermal therapy (PTT) targeting anaplastic thyroid carcinoma (ATC) [112]. The system consists of core–shell spherical nanoparticles composed of CuS nanocrystals coated with BSA. The BSA coating provides multiple functional groups for radiolabeling, including thiol (–SH), amine (–NH_2_), and carboxyl (–COOH) groups. These sites allow for stable radioiodine (^131^I) labeling using the chloramine-T method, which enables the electrophilic substitution of iodine atoms on tyrosine or other reactive residues of BSA. These sub-22 nm nanoparticles demonstrated superior antitumor efficacy in vitro and in vivo compared to monotherapies. SPECT imaging revealed a peak in tumor radioactivity at 24 h post-injection with persistence up to 5 days. The system achieves a photothermal conversion efficiency of 28.07%, and showed no significant toxicity over a 30-day period. Beyond thyroid oncology, albumin nanoparticles show promise in other therapeutic domains, including pulmonary drug delivery, where biodegradability, biocompatibility, and non-inflammatory properties are essential. In this context, Woods et al. evaluated ^111^In-labeled BSA nanoparticles administered via the pulmonary route in mice [113]. The nanosystem consists of self-assembled human serum albumin spherical nanoparticles, prepared using the desolvation method. Radiolabeling was achieved using ^111^In complexed with DTPA, which was covalently attached to albumin via isothiocyanate chemistry. The lysine residues on the albumin surface provided primary amines for DTPA conjugation, enabling stable coordination with ^111^In for SPECT/CT imaging. Administered doses ranged from 2 to 390 µg, and biodistribution was assessed using SPECT/CT imaging. Although no active targeting ligands were used, the albumin nanoparticles exhibited prolonged retention in lung tissue due to their size-dependent deposition and interaction with alveolar macrophages. The particles were mainly cleared via mucociliary transport and translocation across the alveolar barrier, with slow systemic distribution compared to soluble albumin. This passive accumulation in lung tissue makes them suitable for sustained pulmonary drug delivery.

### 4.4. PLGA Nanoparticles

Poly (lactic-co-glycolic acid) (PLGA) nanoparticles have emerged as a highly versatile platform for biomedical applications due to their biocompatibility, biodegradability, and regulatory approval for clinical use. These polymeric carriers can encapsulate a wide range of therapeutic agents, including small molecules, peptides, and radionuclides, allowing for controlled release, enhanced bioavailability, and tumor-targeted delivery. Notably, incorporating radiolabels into PLGA nanoparticles enhances their utility by enabling non-invasive tracking and real-time imaging through modalities such as SPECT and PET [114]. These nanoparticles have been applied to various research fields. Recently, Uygur et al. developed a ^99m^Tc-radiolabeled spherical PLGA formulation encapsulating madecassoside, a plant-derived antioxidant, conjugated with levodopa, for application in Parkinson’s disease (PD) [115]. The radiolabeling efficiency (>95%) was achieved by coordinating ^99m^Tc directly to functional groups within the PLGA-encapsulated MA and MA–L-DOPA structures. Likely coordination sites include hydroxyl and carboxyl groups present in the plant-based molecules and the PLGA matrix, allowing complexation with reduced ^99m^Tc (SnCl_2_ used as a reducing agent). Cytotoxicity assays confirmed concentration-dependent effects on SH-SY5Y and PC-12 neuronal cell lines, with significant midbrain uptake in biodistribution studies, suggesting its promise as a radiodiagnostic probe in PD. In other research fields, such as neuropathic pain, a complex condition associated with nerve injury or disease, intranasal delivery could bypass the blood–brain barrier. Nigam et al. formulated ^99m^Tc-labeled lamotrigine-loaded spherical PLGA nanoparticles for central nervous system targeting [116]. ^99m^Tc labeling was achieved by reducing technetium-pertechnetate using stannous chloride, and allowing it to coordinate with functional groups on lamotrigine and potentially with residual polar groups on PLGA. The system achieved a radiolabeling efficiency of 97%, with a drug targeting efficiency of 130% via the intranasal route. SPECT imaging confirmed superior brain accumulation compared to intravenous and oral routes.

**Figure 7:**
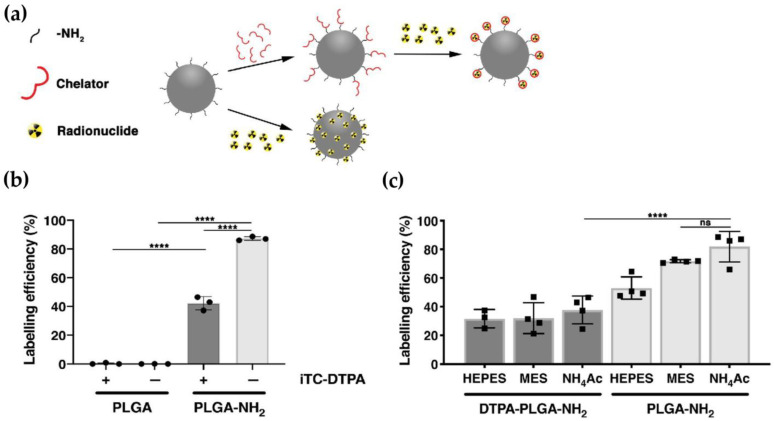
Schematic of the radiolabeling process of PLGA nanoparticles with ^111^In. (**a**) Diagram depicting the proposed mechanisms for radiolabeling PLGA-NH_2_ nanoparticles with and without a chelator; (**b**) comparison of ^111^In-labeling efficiency for PLGA and PLGA-NH_2_ nanoparticles, with and without conjugation to the chelator diethylenetriaminepentaacetic acid (DTPA) (n = 3); (**c**) ^111^In-labeling efficiency of DTPA-conjugated and nonconjugated PLGA-NH_2_ nanoparticles in different media (n = 3–4). Statistical significance: ns = not significant, **** *p* < 0.0001. Reproduced with permission [117].

PLGA nanoparticles have also been employed in immune cell tracking. Krekorian et al. developed spherical PLGA-NH_2_ nanoparticles for the chelator-free ^111^In labeling of THP-1 cells (Figure 7) [117]. Chelator-free radiolabeling with ^111^In was performed via direct coordination of the metal with surface amine groups on PLGA-NH_2_. These modified nanoparticles achieved labeling efficiencies of 87%, compared to <1% for unmodified particles. The system demonstrated excellent in vivo retention and preserved cell viability, allowing the real-time SPECT tracking of immune cell migration in infection models.

### 4.5. Dendrimers

**Figure 8:**
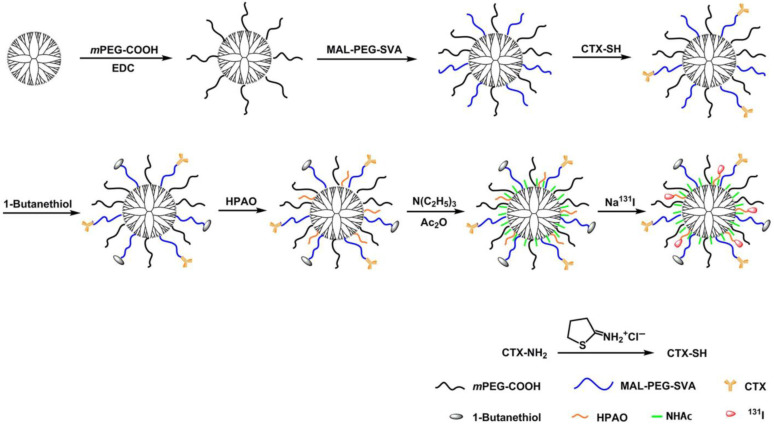
Diagram of the synthesis process for ^131^I-G5.NHAc-HPAO-(PEG-CTX)-(mPEG) dendrimers. Reproduced with permission [122].

Dendrimers are highly branched, monodisperse macromolecules with a tree-like architecture that makes them ideal candidates for biomedical applications, particularly in drug delivery and molecular imaging [118]. The most common dendrimer families include polyamidoamine (PAMAM), poly (propyleneimine) (PPI), polybenzyl ether, polyesteramide, polyaliphatic ester, and polycarbosilane [119]. The versatility of dendrimers enables their use as carriers for both therapeutic and diagnostic agents. Their terminal groups can be easily modified with radionuclide chelators and targeting ligands, such as biotin, folic acid, peptides, aptamers, and monoclonal antibodies. For nuclear imaging, SPECT-compatible isotopes, like ^99m^Tc, ^111^In, and ^125^I, and PET isotopes, such as ^18^F, ^68^Ga, and ^64^Cu, are commonly used [120,121]. For therapeutic purposes, dendrimers have been conjugated with beta emitters (^177^Lu, ^188^Re, ^90^Y), alpha emitters (^213^Bi), and Auger electron emitters (^111^In). The utility of radiolabeled dendrimers for targeted SPECT imaging and radiotherapy has been demonstrated in specific tumor models. Zhao et al. developed G5 PAMAM dendrimers conjugated with chlorotoxin (CTX) and labeled with ^131^I. These spherical dendrimers bearing surface primary amines were modified with HPAO (3-(4′-hydroxyphenyl)propionic acid) for electrophilic ^131^I labeling and with CTX via PEG linkers (Figure 8). CTX specifically targets matrix metalloproteinase-2 (MMP-2) on glioma cells, mediating receptor-driven internalization. The ^131^I-CTX-dendrimers showed superior tumor uptake and therapeutic efficacy in glioma-bearing mice, with no observable systemic toxicity.

Recently, dendrimers have also been used to enhance the biocompatibility of metal-based nanoparticles. Almasi et al. synthesized spherical core–shell Fe_3_O_4_@G4 PAMAM constructs, whose surface primary amines were DOTA-functionalized via p-SCN-Bn-DOTA thiourea linkage for ^68^Ga labeling [123]. These particles achieved 3.4 % ID/g tumor uptake—driven by the enhanced permeability and retention (EPR) effect—alongside prolonged circulation and thermal stability at 60 °C. However, significant liver phagocytosis limited selectivity, indicating a need for additional stealth modifications to evade the reticuloendothelial system.

### 4.6. Gold Nanoparticles

Gold nanoparticles (AuNPs) have emerged as a versatile platform in radionanomedicine due to their unique physicochemical properties, including high biocompatibility, ease of surface modification, and size/shape tunability. These features enable both passive accumulation in tumors via the enhanced permeability and retention (EPR) effect and active targeting through ligand conjugation. Their adaptability to various nanostructures—such as nanospheres, nanorods, nanocages, and nanoshells—makes them suitable carriers for a broad range of diagnostic and therapeutic radionuclides [21]. For example, Chakravarty et al. developed ^198^Au-labeled AuNPs functionalized with RGD through a one-step method [124]. These spherical AuNPs are intrinsically radiolabeled during the c(RGDfK)-mediated reduction in HAuCl_4_, embedding ^198^Au in the gold lattice. The c(RGDfK) shell binds α_v_β_3_ integrins on B16F10 melanoma cells to drive receptor-mediated internalization, yielding a high tumor uptake (8.7 ± 2.1 % ID/g at 4 h) and significant tumor regression without toxicity.

On the other hand, environmentally friendly synthesis methods have gained interest. Sakr et al. synthesized spherical AuNPs via green reduction with gallic acid, whose phenolic groups directly chelate ^99m^Tc [66]. These biocompatible particles accumulate in tumors via the passive EPR effect and show >99% retention after intratumoral injection, with favorable biodistribution for SPECT imaging.

**Figure 9:**
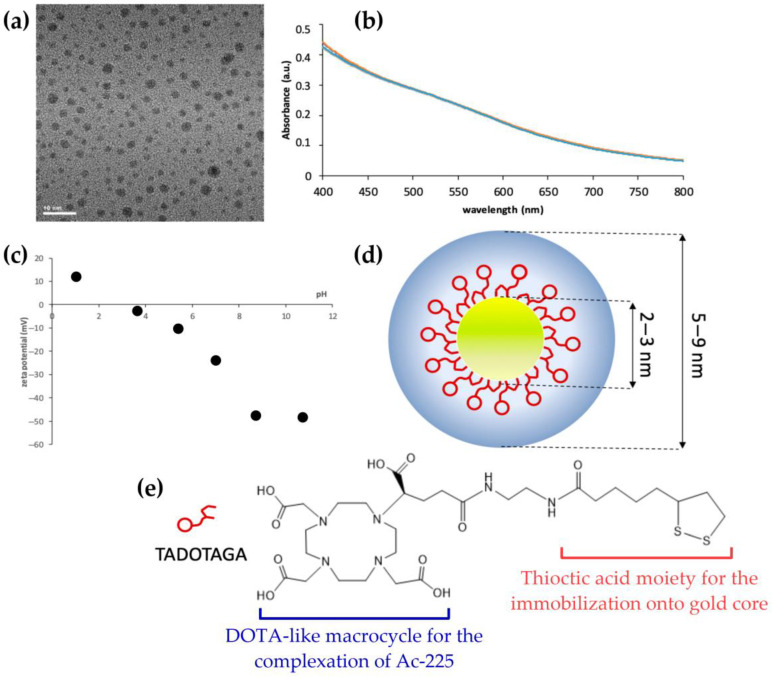
Characterization and structure of Au@TADOTAGA nanoparticles. (**a**) TEM image of Au@TADOTAGA nanoparticles; (**b**) UV-visible spectra measured at pH 3 (blue), 5 (red), 7 (green), 9 (purple), and 11 (cyan); (**c**) zeta potential of Au@TADOTAGA nanoparticle aqueous suspension as a function of pH; (**d**) diagram illustrating an Au@TADOTAGA nanoparticle (yellow: gold core; red: TADOTAGA molecules in the organic shell; blue: immobile solvation layer); (**e**) molecular structure of TADOTAGA. Reproduced with permission [126].

In an effort to address glioma-specific delivery challenges, Zhao et al. designed ^131^I-labeled AuNPs based on polyethylenimine (PEI) and functionalized with chlorotoxin (CTX) for glioma targeting and SPECT/CT imaging [125]. These spherical Au PENPs were radiolabeled via phenolic HPAO groups linked to PEI surface amines, enabling efficient ^131^I electrophilic substitution. The CTX–Au PENPs successfully crossed the blood–brain barrier, accumulated in glioma tissues through MMP-2 binding, and produced potent therapeutic outcomes without adverse effects, reinforcing their potential for CNS applications. In the context of alpha-emitting radionuclides, Salvanou et al. developed a ^225^Ac-labeled AuNP (^225^Ac-Au@TADOTAGA) for intratumoral brachytherapy [126]. These spherical nanoparticles consist of a gold core coated with the macrocyclic TADOTAGA chelator, which enables stable coordination of ^225^Ac (Figure 9). The formulation, administered without active targeting ligands, relies on direct intratumoral injection for local retention (>95 %) and significantly inhibited tumor growth in U-87 MG glioma-bearing mice, highlighting the feasibility of AuNP-based alpha therapy for aggressive tumor phenotypes.

**Figure 10:**
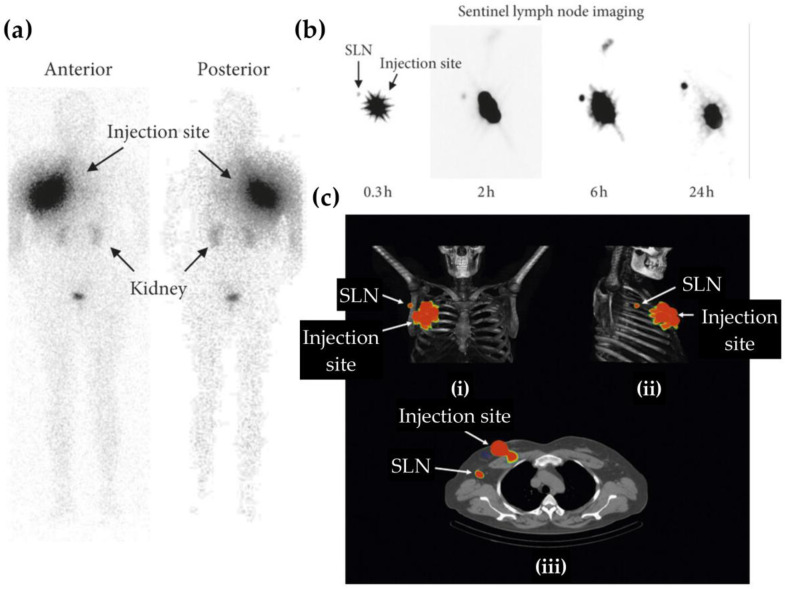
Representative planar and tomographic imaging of a patient following administration of ^99m^Tc-AuNP-mannose (37 MBq). (**a**) Anterior and posterior whole-body planar images acquired 2 h post-injection, showing predominant renal clearance, (**b**) frontal planar views of the breast region at successive time points, highlighting the injection site and sentinel lymph node, (**c**) SPECT/CT imaging 6.5 h post-administration, frontal (**i**), lateral (**ii**), and fused SPECT/CT slice depicting ^99m^Tc-AuNP-mannose localization within the sentinel lymph node (**iii**). Reproduced with permission [127].

Finally, at a translational milestone, Ramírez-Nava et al. reported the first-in-human study using ^99m^Tc-AuNP-mannose constructs for sentinel lymph node (SLN) mapping in breast cancer [127]. These spherical AuNPs (~20 nm) were radiolabeled through HYNIC groups present on mannose-bearing peptides, enabling direct and stable coordination of ^99m^Tc without additional chelators, while the mannose ligands actively targeted macrophage receptors within SLNs. The formulation selectively accumulated in SLNs, exhibited no migration to non-target nodes, and demonstrated excellent safety with an effective dose of 2.1 μSv/MBq, supporting clinical feasibility (Figure 10).

### 4.7. Iron Oxide Nanoparticles

Magnetic iron oxide nanoparticles (IONPs) have emerged as a powerful tool in nanomedicine due to their unique magnetic properties and biocompatibility. Their core compositions—commonly magnetite (Fe_3_O_4_) or maghemite (γ-Fe_2_O_3_)—enable their use in magnetic resonance imaging (MRI), targeted drug delivery, and hyperthermia therapy. When functionalized with polymers or biological ligands, IONPs can achieve extended systemic circulation, enhanced tumor targeting, and minimal off-target effects. Moreover, when radiolabeled with diagnostic or therapeutic isotopes, IONPs transcend their conventional roles to serve as hybrid agents for dual- or tri-modal imaging and as potent vehicles for targeted radiotherapy [28,128]. IONPs are categorized by size into micrometer-scale paramagnetic iron oxide (MPIO), superparamagnetic iron oxide (SPIO), ultrasmall SPIO (USPIO), and monocrystalline iron oxide nanoparticles (MION). Clinically, they have been used as MRI contrast agents, particularly for imaging the liver, spleen, and bone marrow [129,130]. Their potential expands when radiolabeled with diagnostic or therapeutic isotopes, enabling hybrid PET/MRI or SPECT/MRI modalities and positioning IONPs as promising theranostic platforms [131].

**Figure 11:**
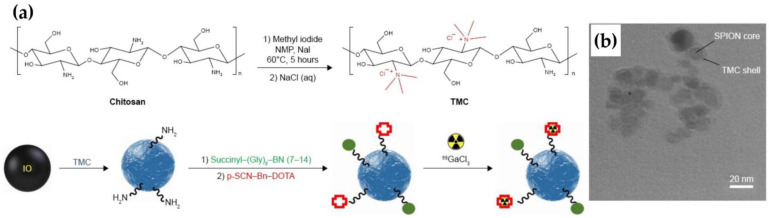
Synthesis and imaging of DOTA–BN–TMC–MNPs. (**a**) Diagram depicting the radiolabeling process of DOTA–BN–TMC–MNPs; (**b**) TEM image of the DOTA–BN–TMC–MNPs. Reproduced with permission [69].

In this context, several recent studies have explored innovative applications of IONPs in both diagnosis and therapy. For instance, Syu et al. designed NIR-absorbing IO nanocrystals that enabled photothermal therapy [132]. These spherical aggregates were composed of ultrasmall Fe_3_O_4_ cores, synthesized by ligand-assisted co-precipitation. Although not radiolabeled, their surfaces presented amine and carboxyl groups (from dopamine derivatives) with potential for future chelation. The nanocrystals showed no cytotoxicity in the absence of laser activation but effectively ablated tumors under NIR irradiation, with enhanced MR contrast and magnetic field-guided accumulation in tumor tissue, confirming their biocompatibility and therapeutic performance. Similarly, in the field of breast cancer imaging, Hajiramezanali et al. developed ^68^Ga-labeled SPIONs functionalized with bombesin and coated them with N,N,N-trimethyl chitosan [69]. These spherical SPIONs were radiolabeled via DOTA chelators conjugated to surface amines on the TMC shell (Figure 11). The bombesin ligand actively targeted gastrin-releasing peptide receptors (GRPr) on T-47D breast cancer cells, resulting in increased tumor uptake in PET/CT imaging and favorable tumor-to-background ratios, highlighting their utility in GRPr-targeted imaging.

Furthermore, to address challenges related facing systemic clearance and improve intratumoral retention, Stanković et al. proposed the use of ^177^Lu-labeled SPIONs for nanobrachytherapy [133]. These spherical DMSA@SPIONs were radiolabeled through surface thiol groups provided by the DMSA coating, enabling stable coordination of ^177^Lu under mild conditions. The formulation relies on direct intratumoral injection for localized delivery and retention. Following administration, ^177^Lu-DMSA@SPIONs significantly inhibited tumor growth in colorectal and mammary tumor models with minimal systemic leakage, suggesting a safe and effective alternative to conventional brachytherapy.

### 4.8. Silica Nanoparticles

Silica nanoparticles (SiNPs) have emerged as highly promising candidates in the realm of nanomedicine, owing to their biocompatibility, chemical stability, and structural tunability. Composed of materials naturally present in the human body, such as silicon dioxide, SiNPs are generally recognized as safe (GRAS) by the U.S. Food and Drug Administration [134]. Unlike other inorganic nanoparticles, silica itself does not exhibit intrinsic imaging or therapeutic functions. Nonetheless, its excellent physicochemical properties make it an ideal carrier for delivering imaging agents, therapeutic radionuclides, and chemotherapeutic drugs. Advances in nanotechnology have led to the development of diverse silica-based architectures, including mesoporous, hollow, and ultrasmall variants, which have been exploited for both diagnostic and therapeutic applications, particularly in oncology [42]. Various architectures of SiNPs have been developed, including dense silica (dSiO_2_), mesoporous silica nanoparticles (MSNs), biodegradable MSNs, hollow mesoporous silica (HMSNs), and ultrasmall fluorescent core–shell Cornell prime dots (C′ dots) [112,135]. These platforms allow for surface modification with chelators, targeting ligands, or other nanoparticles (e.g., Au, Fe_3_O_4_, WO_3_) to achieve multimodal functionality. Radiolabeled SiNPs can thus support diagnostic (PET/SPECT) and therapeutic (β-/α-emitters) applications in oncology and other fields.

In one of the earliest and most prominent demonstrations of these capabilities, Benezra et al. synthesized ultrasmall fluorescent silica nanoparticles (C′ dots) encapsulating Cy5 and radiolabeled with ^124^I [136]. These silica nanoparticles featured a dye-doped core, a thin silica shell, and surface silanol groups that enabled efficient radiolabeling via iodogen-mediated electrophilic substitution. Functionalized with PEG and the integrin-targeting cyclic RGD peptide, these 7 nm particles demonstrated >95% radiochemical yield and selective accumulation in α_v_β_3_-positive melanoma xenografts. PET imaging revealed prolonged tumor retention and favorable tumor-to-blood ratios. Comparisons with non-targeted controls (^124^I-PEG-dots) confirmed the enhancement provided via molecular targeting. In a separate line of investigation, Kamkaew et al. introduced an innovative approach to photodynamic therapy (PDT) by using ^89^Zr-labeled HMSNs loaded with chlorin e6 [137]. These spherical HMSNs feature a central cavity and uniform mesopores for encapsulating photosensitizers. Radiolabeling with ^89^Zr is achieved via coordinative binding to the oxophilic silanol groups on the silica matrix, while Ce6 is loaded through hydrophobic and π–π interactions. Following subcutaneous injection, the nanoparticle accumulates in tumors via passive retention, where Cerenkov radiation from ^89^Zr serves as the excitation source, enabling PDT without external light. Significant tumor growth inhibition was observed in murine models, validating the in vivo feasibility of Cerenkov radiation-induced PDT.

**Figure 12:**
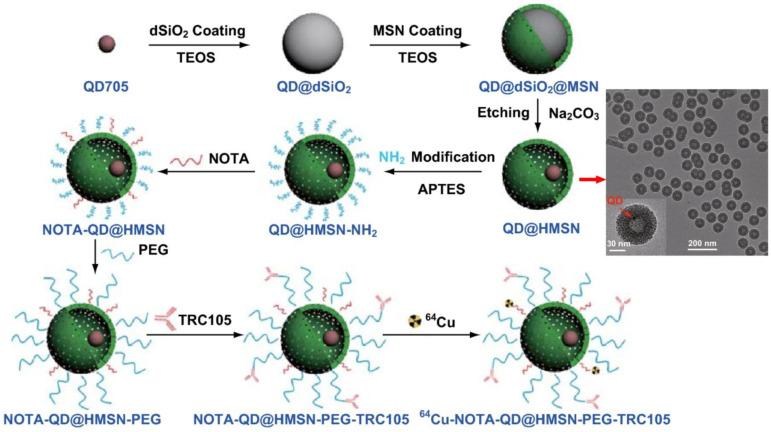
Synthesis and imaging of QD@HMSN yolk/shell nanosystem. Diagram showing the synthesis and functionalization process of the QD@HMSN yolk/shell-structured silica nanosystem, with red arrows indicating a TEM image of the QD@HMSN. Reproduced with permission [138].

Further illustrating the versatility of SiNPs, Shi et al. designed a core-shell silica nanoplatform (QD@HMSN) containing NIR quantum dots and radiolabeled with ^64^Cu [138]. This yolk/shell structure consists of a quantum dot core encapsulated within a hollow mesoporous silica shell, while radiolabeling was achieved by grafting NOTA chelators onto aminated silanol surfaces, enabling stable ^64^Cu coordination (Figure 12). Surface functionalization with PEG and TRC105 antibody facilitated specific binding to tumor endothelium. This multimodal system enabled PET and optical imaging while specifically targeting tumor vasculature. It also enhanced doxorubicin delivery via pH-responsive release mechanisms. PET imaging showed a 1.5-fold increase in tumor accumulation, and histological analyses confirmed improved delivery and specificity.

Beyond oncology applications, Polyák et al. applied silica-based magnetic nanoparticles to address periprosthetic joint infections (PJI), a major complication in orthopedic surgery [139]. These ~100–150 nm particles comprise superparamagnetic iron oxide cores encased in a porous silica shell functionalized with THP chelators for efficient ^68^Ga labeling. PEGylation reduced hepatic uptake, while systemic administration enabled moderate accumulation at subcutaneous magnetic implants, supporting non-invasive PET/CT imaging of infection sites and highlighting challenges in local delivery.

**Figure 13:**
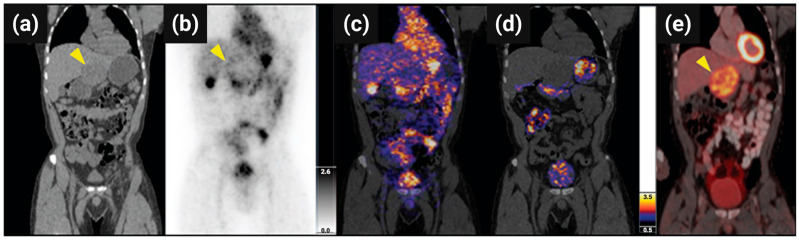
Whole-body PET-CT imaging of a patient with anorectal mucosal melanoma and a known left hepatic lobe metastasis following intravenous administration of ^124^I-cRGDY–PEG–C dots. (**a**) Reformatted coronal CT acquired 4 h post-injection demonstrating a hypodense lesion in the inferior left hepatic lobe (yellow arrowhead), (**b**) coronal PET image at 4 h post-injection showing particle uptake circumscribing the tumor margin (yellow arrowhead), as well as activity in the bladder, gastrointestinal tract, gallbladder, and heart, (**c**) co-registered PET-CT at 4 h, confirming particle localization to the tumor margin, (**d**) co-registered PET-CT at 24 h, indicating clearance of particle activity from the tumor, and (**e**) corresponding ^18^F-FDG PET-CT image, revealing a hypermetabolic tumor border in the hepatic metastasis (yellow arrowhead), reflecting glucose metabolism. Reproduced with permission [140].

Finally, Phillips et al. conducted the first-in-human study of ^124^I-cRGDY-PEG-C′ dots in patients with metastatic melanoma (NCT01266096) (Figure 13) [140]. These ultrasmall (~6 nm) amorphous silica nanoparticles demonstrated excellent safety, rapid renal clearance, and minimal liver accumulation (~1.28 × 10^−3^ %ID/g at 72 h), in contrast to conventional nanoparticles. However, although specific uptake in certain lesions was observed (e.g., ~0.01% of the injected dose in a pituitary microadenoma), overall tumor accumulation was relatively low compared to ^18^F-FDG, as also noted by the authors. This limited tumor uptake may be attributed to the low injected mass and the microdosing design of the trial, which was not optimized for maximizing lesion visualization. These findings support the clinical potential of ultrasmall SiNPs for molecular imaging and underscore their relevance in advancing personalized nanomedicine. Subsequent trials explored C′ dots for PET imaging of brain cancers and pituitary adenomas using ^89^Zr-DFO-cRGDY-PEG-Cy5-C′ dots (NCT03465618). Another ongoing study employs ^64^Cu-NOTA-PSMA-PEG-Cy5.5-C′ dots to guide intraoperative prostate cancer imaging (NCT04167969), aiming to improve surgical precision via PET/MRI fusion.

### 4.9. Comparative Advantages of Nanoradiopharmaceuticals over Conventional Radiopharmaceuticals: Preclinical and Clinical Evidence

Radiolabeled nanoparticles demonstrate significant clinical advantages over conventional radiopharmaceuticals, as evidenced by preclinical models and early-phase human trials. These benefits, supported by comparative pharmacokinetic and therapeutic data, highlight the translational potential of nanostructured systems for imaging and therapy.

In clinical settings, PEGylated liposomal constructs, such as ^64^Cu-MM-302, exhibit enhanced tumor retention and variable uptake correlating with therapeutic response in HER2-positive breast cancer patients. A first-in-human PET/CT study of MM-302 revealed 35-fold interpatient variability in tumor accumulation, suggesting its utility as a predictive biomarker for personalized treatment, a capability less achievable with small-molecule radiotracers.

For molecular imaging of melanoma, ultrasmall PEGylated silica nanoparticles (C′ dots) radiolabeled with ^124^I show rapid renal clearance and minimal hepatic uptake, addressing a key limitation of conventional agents that often accumulate in the liver and spleen. In metastatic melanoma patients, these nanoprobes achieved high tumor-to-background contrast, validating their clinical potential. Ongoing trials are exploring their application in brain and prostate cancers, including image-guided surgery.

Preclinical studies further confirm the superior therapeutic indices and targeting precision of nanoradiopharmaceuticals. For instance, ^131^I-labeled BSA@CuS nanoparticles demonstrated prolonged tumor retention and suppression for up to five days in murine anaplastic thyroid carcinoma models, outperforming free iodine treatments. Similarly, micellar formulations co-delivering ^188^Re and docetaxel exhibited synergistic chemoradiotherapeutic effects and extended circulation, while ^99^ᵐTc-functionalized micelles enabled non-invasive CNS delivery via the intranasal route, a pathway largely inaccessible to conventional radiopharmaceuticals.

These findings underscore the distinct advantages of nanoradiopharmaceuticals, including the following: (i) enhanced radionuclide payload capacity, (ii) improved tumor-to-background ratios, (iii) optimized pharmacokinetics and prolonged target-site retention, (iv) integration of multimodal functionalities, and (v) superior safety and clearance profiles in clinical settings. These attributes position nanoradiopharmaceuticals as a multifunctional platform for enhanced diagnostic accuracy, therapeutic efficacy, and precision medicine in nuclear imaging and radiotherapy.

## 5. Discussion

The integration of nanotechnology with nuclear medicine has introduced a transformative paradigm in radiopharmacy, wherein coupling radionuclides to nanoscale carriers markedly enhances specificity, sensitivity, multifunctionality, and safety compared to conventional small-molecule tracers. By exploiting the EPR effect together with active targeting moieties, these nanoradiopharmaceuticals achieve more efficient tumor accumulation and prolonged retention, resulting in elevated tumor-to-background contrast in SPECT and PET scans, as well as more precise therapeutic dose delivery. A critical determinant of these advances lies in the strategic selection of radionuclide—based on parameters such as half-life, emission type (α, β, or Auger electrons), linear energy transfer, and decay characteristics—and their optimal integration with nanoparticle properties. Extrinsic radiolabeling using bifunctional chelators like DOTA or NOTA affords broad compatibility with various radiometals and design flexibility. Conversely, intrinsic labeling approaches—including co-synthesis of radioactive and stable precursors, specific trapping, cation exchange, or proton beam activation—offer enhanced in vivo stability and reduced risk of radiometal dissociation.

A diverse array of nanoparticle platforms has demonstrated efficacy in preclinical and early clinical studies. Liposomes—like the ^64^Cu-labeled MM-302—exemplify the utility of predictive imaging biomarkers and theranostic feedback loops, with tumor uptake correlating with treatment response in breast cancer. Micellar systems that co-deliver chemotherapy and β-emitters (for example, ^188^Re-docetaxel micelles) highlight the potential of synergistic chemoradiotherapy, while intranasal delivery of ^99m^Tc-tagged micelles has enabled blood–brain barrier penetration for glioblastoma targeting, resulting in enhanced cytotoxicity. PLGA nanoparticles extend these functionalities to neural and immune cell tracking, facilitating dynamic SPECT/CT imaging of therapeutic biodistribution. Dendrimers—monodisperse, highly branched polymers—offer high chelator-loading capacities for both diagnostic and α- or β-particle therapeutic applications. Gold nanoparticles, with tunable surface chemistry and morphology, function effectively as passive EPR agents or actively targeted vectors for radiotherapies involving ^198^Au and ^225^Ac, including successful use in sentinel lymph node mapping in clinical settings. Meanwhile, iron oxide nanoparticles labeled with isotopes like ^68^Ga, ^64^Cu, or ^99m^Tc have enabled dual-modality PET/MRI or SPECT/MRI applications. When functionalized with ^177^Lu-DMSA@SPIONs, these agents have shown potent antitumor effects through intratumoral nanobrachytherapy, with minimal off-target distribution and systemic toxicity.

Silica-based nanocarriers—ranging from dense and mesoporous particles to hollow architectures and ultrasmall core–shell “C′ dots”—exemplify the versatility of these platforms. Notably, first-in-human trials of ^124^I-cRGDY-PEG-C′ dots in patients with metastatic melanoma demonstrated rapid renal clearance and minimal hepatic uptake, validating their favorable safety profile. Ongoing studies with ^89^Zr-DFO-cRGDY-PEG-Cy5-C′ dots for brain and pituitary imaging and ^64^Cu-NOTA-PSMA-PEG-Cy5.5-C′ dots for intraoperative guidance in prostate cancer surgery. These applications aim to enhance surgical precision and support real-time dosimetry via PET/MRI image fusion.

Despite these promising results, bringing nanoradiopharmaceuticals into standard clinical use remains challenging. Here, we outline several critical challenges that must be addressed in future research. First, we must achieve truly uniform nanoparticles—consistent in size, shape, and composition—backed by thorough physicochemical characterization to guarantee reproducible behavior [3]. Biocompatibility is equally vital, requiring materials that avoid toxicity and unwanted immune reactions. At the same time, fine-tuning biodistribution and pharmacokinetics remains a challenge, as many nanoparticles end up trapped in the liver, spleen, or kidneys, rather than homing in on their targets, and attaching targeting ligands to short-lived radionuclides only adds to the complexity [21,141]. On the regulatory front, clearer guidelines are needed to navigate the evolving approval landscape for nanomedicines, and scalable, reliable manufacturing processes must be developed to transition these agents from the lab to the clinic [142,143]. Ensuring long-term stability—preventing aggregation or degradation during storage and handling—also demands careful formulation strategies. Looking forward, exciting avenues include designing single nanoconstructs that combine diagnostic and therapeutic functions for true multimodal theranostics, employing image-guided radiation therapy with on-site activation of nanoparticles, and customizing treatments to each patient’s unique biology [144]. Advances in stimuli-responsive materials that react to pH, temperature, or light, along with optimized ligand presentation to boost targeting efficiency, plus combination therapies that pair nanoradiopharmaceuticals with chemotherapy or immunotherapy, and efforts to sharpen imaging resolution, promise to bring us closer to safer, more precise, and more effective cancer diagnosis and treatment.

Looking ahead reveals that the integration of artificial intelligence in formulation design, microfluidic production technologies, and advanced imaging analytics is poised to expedite the development of next-generation nanoradiopharmaceuticals. Emerging multifunctional constructs, featuring stimuli-responsive components, biomimetic coatings, and site-specific release mechanisms, offer enhanced selectivity for tumor targeting and improved therapeutic indices. These innovations hold promise for further personalizing treatment regimens and advancing the clinical translation of precision radiotheranostics.

## 6. Future Outlooks

The continued advancement of nanoradiopharmaceuticals hinges on tackling key challenges while harnessing their unique therapeutic and diagnostic benefits. These multifunctional platforms provide superior specificity, reduced toxicity, and the potential for integrating imaging, treatment, and monitoring into a single system. Overcoming barriers to clinical translation, such as manufacturing scalability and regulatory validation, is critical for realizing their full potential. Meanwhile, emerging technologies like microfluidics and artificial intelligence are expected to accelerate development and boost precision. This section explores future directions in efficacy, cost-effectiveness, and deep learning integration in nanoradiotheranostics.

Nanoparticle-based drug delivery systems, pioneered by Ehrlich’s “magic bullet” concept [145], hold immense promise for theranostic applications, blending diagnosis and therapy. Radiolabeled multifunctional nanoplatforms offer enhanced specificity, reduced toxicity, and the potential to combine imaging, treatment, and monitoring in one system [108]. Thanks to their small size, high surface area to volume ratio, and tunable chemistry, these nanocarriers enable precise drug delivery and improved cellular uptake compared to larger macromolecules [12,146]. Surface modifications can extend bloodstream circulation and boost accumulation in targets like solid tumors, increasing efficacy and bioavailability while minimizing side effects [63,108]. These nanosystems navigate fine capillaries and lymphatic endothelium, enhancing targeting and prolonging circulation [147]. As a result, they outperform most conventional imaging and theranostic methods, making them a key platform for researching diseases like cancer, neurological disorders, and infections caused by multidrug-resistant bacteria [148].

Despite their potential, moving these nanoplatforms from preclinical studies to clinical use has been slow due to challenges in consistent, scalable production [149]. These hurdles raise initial costs but can be addressed by adopting standardized preclinical validation systems and good manufacturing practices (GMPs) to ensure safety and efficacy [108,150]. Microfluidic technology, for instance, could reduce batch variations and improve production efficiency, speeding up clinical translation [145]. Such advancements could make the benefits of precise diagnostics, personalized treatment, and fewer side effects outweigh the initial costs, ultimately enhancing patient quality of life by reducing toxicity and improving treatment efficacy [151].

Deep learning (DL) further amplifies these advancements by enhancing medical imaging and nanoplatform design [152]. Convolutional neural networks, generative adversarial networks, and diffusion models improve tumor detection and image quality in PET and SPECT scans, restoring diagnostic accuracy in low-dose scans with high sensitivity and specificity [153,154,155]. DL also enables synthetic image generation, improving patient safety without compromising reliability [156,157].

In nanomedicine, machine learning (ML) and DL tools like NanoMask automated 3D segmentation and pharmacokinetics analysis in PET/CT datasets, achieving over 92% accuracy in tumor uptake quantification [158,159,160,161,162]. Moreover, neural network-based tools facilitate the rational design of nanotheranostics by streamlining nanoparticle synthesis and characterizing nano–bio interactions [163]. In preclinical models, DL has also been used to assess nanoparticle uptake in tumor cells via fluorescence imaging, providing a high-throughput evaluation of targeting strategies [164]. Together, these ML/DL applications could help bridge the gap between nanoformulation design and clinical imaging, offering data-driven guidance to enhance the efficacy, safety, and personalized deployment of nanoradiopharmaceutical platforms.

## 7. Conclusions

In conclusion, nanoradiopharmaceuticals increase the strategies or tools for personalized medicine. By integrating nanotechnology with nuclear medicine, researchers have developed nanoscale constructs capable of selectively targeting tumors and delivering or enabling therapy within a single platform. This dual functionality facilitates the real-time monitoring of therapeutic response and enables dynamic, patient-specific treatment adjustments. Furthermore, the incorporation of radioactive isotopes into these nanostructures enhances their in vivo stability, minimizes off-target effects, prolongs circulation times, and promotes preferential tumor accumulation. As this field continues to evolve, nanoradiopharmaceuticals hold transformative potential for nuclear medicine, offering a path toward broader clinical adoption of nanotechnology and more precise, effective care for patients.

## Figures and Tables

**Table 1:** Comparative table summarizing the key characteristics of beta, alpha, and Auger electron emitters [2,12,16,17,40,88,89,90,91].

Property	Beta Emitters	Alpha Emitters	Auger Electron Emitters
Kinetic Energy (MeV)	0.3–2.3	2–10	0.003–0.04
Penetration Range in Tissue	0.5–12 mm	50–80 µm	1–500 nm
Linear Energy Transfer (LET)	0.2–2 keV/μm	~100 keV/μm	4–26 keV/μm
Main Mechanism	Crossfire & bystander effects	Localized, high-energy DNA damage (double-strand breaks)	Highly localized ionization near DNA
Biological Effect	Damage to both targeted and nearby cells	Irreparable DNA damage in targeted cells	Comparable to alpha particles but at nanometric scale
Example Radionuclides	^177^Lu, ^90^Y, ^188^Re, ^64^Cu, ^131^I	^225^Ac, ^213^Bi, ^211^At, ^212^Pb/^212^Bi, ^223^Ra, ^149^Tb	^125^I, ^111^In, ^99^ᵐTc
Nanoplatform Strategies	MOFs (e.g., MIL-101 for ^188^Re), PLGA NPs (^177^Lu), chelator-free hydrogels	Ag_2_TeNPs for ^212^Pb/^212^Bi, Au-RGD NPs for ^211^At, nHA for ^223^Ra for bone targeting	TiO_2_ nanocatalysts with ^125^I, nanobranchytherapy systems
Advantages	Broad tumor coverage, good for heterogeneous/multicellular tumors	Highly cytotoxic to small/micrometastatic clusters, minimal off-target damage	Exceptional subcellular precision, ideal for nucleus-targeting therapies
Limitations	Lower LET, possible off-target effects if not well-confined	Limited range requires precise targeting, decay chain considerations	Extremely short range demands precise nuclear localization, challenging delivery
Clinical or Preclinical Uses	Ovarian cancer (^177^Lu-PLGA), injectable brachytherapy hydrogels	Bone metastases (^223^Ra-nHA), integrin-targeting (^211^At-Au NPs), in situ decay (^212^Pb → ^212^Bi)	Pancreatic cancer (ROS generation via ^125^I-TiO_2_), breast/prostate cancers (targeted nanobranchytherapy)

## Data Availability

This review summarized references from the literature, and no new data were created.
